# Pain-induced sleep disturbances fully mediate the association between symptomatic hip and knee osteoarthritis and poor sleep quality: a cross-sectional study

**DOI:** 10.1302/2633-1462.73.BJO-2025-0325.R1

**Published:** 2026-03-12

**Authors:** Juliette C. Sorel, Bowien H. W. Korterink, Raymond Noordam, Frits R. Rosendaal, Magreet Kloppenburg, Rob G. H. H. Nelissen, Saskia le Cessie, Rudolf W. Poolman, Birit F. P. Broekman, Maaike G. J. Gademan, On behalf of the LOAS and NEO study group

**Affiliations:** 1 Department of Orthopaedics, Leiden University Medical Centre, Leiden, Netherlands; 2 Department of Orthopaedics, Diakonessenhuis, Utrecht, Netherlands; 3 Department of Clinical Epidemiology, Leiden University Medical Centre, Leiden, Netherlands; 4 Health Campus the Hague/Public Health and Primary Care, Leiden University Medical Centre, the Hague, Netherlands; 5 Department of Rheumatology, Leiden University Medical Centre, Leiden, Netherlands; 6 Department of Biomedical Data Sciences, Leiden University Medical Center, Leiden, Netherlands; 7 Department of Orthopaedic and Trauma Surgery, Joint Research, OLVG, Amsterdam, Netherlands; 8 Department of Psychiatry and Medical Psychology, OLVG, Amsterdam, Netherlands; 9 Department of Psychiatry, Amsterdam UMC location Vrije Universiteit Amsterdam, Amsterdam, Netherlands; 10 Amsterdam Public Health, Mental Health program, Amsterdam, Netherlands

**Keywords:** Hip osteoarthritis, Knee osteoarthritis, Sleep quality, Pain, hip and knee osteoarthritis, hip and knee osteoarthritis, hip, Pittsburgh Sleep Quality Index, Obesity, regression analyses, Epidemiology, pain management, arthritis, comorbidities

## Abstract

**Aims:**

We assessed whether symptomatic end-stage hip and knee osteoarthritis (OA) are associated with poorer sleep quality and to what extent pain mediates these associations.

**Methods:**

We included symptomatic end-stage hip- and knee OA participants from the Longitudinal Leiden Orthopaedics Outcomes of Osteo-arthritis Study (LOAS) and participants without OA from the Netherlands Epidemiology of Obesity (NEO) study. We assessed sleep with the Pittsburgh Sleep Quality Index (PSQI) and performed linear regression analyses to investigate the associations between OA and sleep and the potential mediating effects of pain-related sleep disturbances.

**Results:**

Overall, 54% of the 922 hip OA and 48% of the 870 knee OA patients reported poor sleep (total PSQI > 5), compared with 21% of the 1,165 participants without OA. Both hip and knee OA were associated with worse subjective sleep quality (adjusted difference: 0.37 points (95% CI 0.29 to 0.44) and 0.23 points (95% CI 0.15 to 0.32), respectively) and pain-related sleep disturbances (adjusted difference: 1.75 points (95% CI 1.64 to 1.86) and 1.50 points (95% CI 1.38 to 1.62), respectively). The association of hip and knee OA and worse subjective sleep quality was fully mediated by pain-related sleep disturbances (112% (95% CI 90 to 145) and 123% (95% CI 90 to 191), respectively).

**Conclusion:**

Symptomatic end-stage hip and knee OA are strongly associated with worse sleep quality, which is fully mediated by pain-related sleep disturbances. While the cross-sectional design limits causal inferences, these findings underscore the importance of improving both sleep quality and pain management strategies in symptomatic end-stage OA patients. Addressing sleep disturbances, which are often overlooked in clinical practice, could significantly enhance overall health and quality of life of patients with end-stage hip- or knee OA.

Cite this article: *Bone Jt Open* 2026;7(3):348–356.

## Introduction

Osteoarthritis (OA) is one of the most common musculoskeletal disorders in the world with lifetime incidence rates of 25%^1^ and 45%^2^ for developing either hip or knee OA, respectively. Moreover, the prevalence of OA is expected to increase due to the growing population of older people and incidence of obesity.^3,4^ Total joint replacment (TJR) is the treatment of choice in patients with severe end-stage hip and knee OA when conservative treatments fail to induce sufficient pain relieve and to improve patient daily functioning.^5,6^

Sleep plays a vital role in maintaining overall health and wellbeing.^7,8^ Hence, inappropriate sleep duration and poor sleep quality are both associated with lower general health, mental health, and quality of life.^7,8^ Sleep disturbances are common in OA patients,^9^ with studies showing higher rates in OA patients and associations between pain severity and sleep quality. However, existing research has limitations: most studies combined hip and knee OA despite their potentially distinct effects on sleep,^9^ did not compare with participants without OA,^9-11^ and few focus specifically on end-stage OA.^12^ Regional differences in sleep patterns across Europe, Asia, and the USA highlight the need for region-specific studies,^13^ while European population-based research on end-stage hip and knee OA and sleep is notably lacking.^9,11,14,15^ Moreover, understanding sleep quality in end-stage hip and knee OA patients separately could help improve quality of life through more targeted interventions addressing the sleep-pain relationship in this vulnerable population.

The objective of our study was to compare the quality of sleep of a large group of patients with symptomatic end-stage hip and knee OA (prior to total knee or total hip arthroplasty) to individuals without OA. Additionally, we assessed if the association of self-reported symptomatic end-stage hip and knee OA on self-reported subjective sleep was mediated by trouble sleeping due to pain. We hypothesized that the symptomatic end-stage hip and knee OA patients have worse quality of sleep compared with the population without OA and that this relation is mediated by trouble sleeping due to pain.

## Methods

### Study design

With this cross-sectional study, we compared the quality of sleep using the Pittsburgh Sleep Quality Index (PSQI)^16^ scores of participants of a cohort of Dutch end-stage hip and knee OA patients from the Longitudinal Leiden Orthopaedics Outcomes of Osteo-arthritis study (LOAS)^17^ with the sleep quality of participants without OA of a cohort representing the general Dutch population, the Netherlands Epidemiology of Obesity (NEO) study.^18^

### Participants

The LOAS is a multicentre, longitudinal prospective study examining end-stage OA patients awaiting total hip arthroplasty (THA) and total knee arthroplasty (TKA) in the Netherlands. The overall aim of the LOAS is to investigate mid- and long-term outcomes after arthroplasty and to determine which factors influence outcomes of these joint arthroplasty surgeries.^19^ We included LOAS participants on the waiting list for THA or TKA (i.e. symptomatic end-stage OA) in 2022 and 2023 in two of these hospitals (Alrijne Hospital and OCON Orthopaedic Clinic) as in those hospitals we integrated the PSQI in the survey. Patients were eligible for the LOAS if they were scheduled for primary THA and TKA because of end-stage OA, able to complete Dutch questionnaires on paper or electronically, and aged over 18 years. Patients were excluded if no informed consent was provided, if Dutch language skills were insufficient, or if the patient was scheduled for revision surgery.^19^ Additionally, for this study, patients were excluded if no PSQI data were available.

The NEO is a Dutch population-based prospective cohort study that investigates pathways that are responsible for obesity-related diseases.^18^ The NEO included individuals from 45 to 66 years old between 2008 and 2012 with an oversampling of people with high BMI. Therefore, the NEO study contains data on people with higher BMI, as well as a large cohort of participants representing the general Dutch population, also known as the Leiderdorp cohort.^18^ To attain sleep data of participants without OA, we used data of the NEO Leiderdorp cohort. Of them, we excluded participants from our study if they had no PSQI data; if they had knee OA according to American College of Rheumatology (ACR) criteria or the American College of Rheumatology guidelines or any form of self-reported OA;^20^ or answered ‘yes’ to the question regarding whether they had any form of OA.

### Participant characteristics

We included the following participants characteristics: age, sex, BMI, smoking, living status (alone or with others), and presence of a physical comorbidity. Physical comorbidity was defined as the presence of a self-reported history of either cancer, hypertension, diabetes, or a musculoskeletal disease other than OA.

**Fig. 1:**
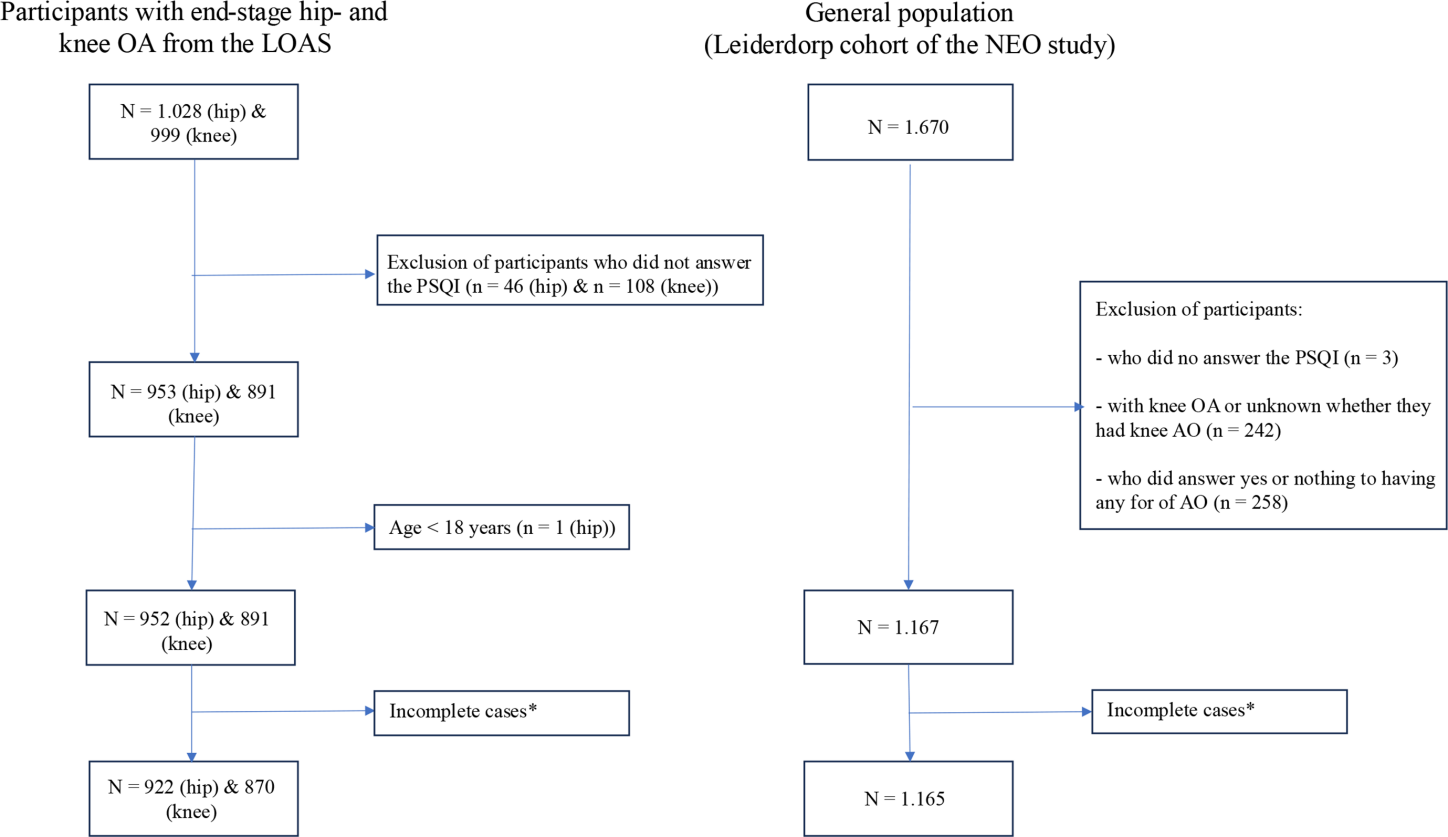
Participant flow diagram. LOAS, Longitudinal Leiden Orthopaedics Outcomes of Osteo-arthritis study; NEO, Netherlands Epidemiology of Obesity; OA, osteoarthritis; PSQI, Pittsburgh Sleep Quality Index.

The total study population consisted of 922 end-stage hip OA patients, 870 end-stage knee OA patients, and 1,165 participants in the control group (Figure 1).

The mean age and BMI were higher in the symptomatic end-stage hip end knee OA groups (Table I). Aditionally, in these OA groups the proportion of females was higher, individuals more often lived alone, and the proportion of participants reporting a comorbidity was higher. Conversely, in the cohort with participants without OA, people tended to smoke more frequently (Table I).

**Table I.** Baseline characteristics of end-stage hip osteoarthritis (OA), end-stage knee OA, and Netherlands Epidemiology of Obesity participants without OA.

Variable	End-stage hip OA (n = 922)	End-stage knee OA (n = 870)	Without OA (n = 1,165)
Mean age, yrs (SD)	67.8 (9.4)	67.9 (8.2)	55.1 (6.1)
Female sex, n (%)	555 (60.2)	479 (55.1)	604 (51.8)
Mean BMI, kg/m^2^ (SD)	27.1 (4.4)	29.1 (4.8)	26.0 (4.3)
Smoking, n (%)	56 (6.1)	41 (4.7)	175 (15.0)
**Living state, n (%)**			
Alone	192 (20.1)	165 (19.0)	137 (11.7)
With others	782 (79.0)	703 (80.8)	1,008 (86.5)
Comorbidity, n (%)*	347 (37.6)	371 (42.6)	345 (29.6)

* Comorbidity includes history of diabetes, hypertension, cancer, or musculoskeletal disease other than osteoarthritis.

### Sleep assessment

We assessed the quality of sleep with the PSQI. The PSQI is a well-known validated questionnaire evaluating self-reported sleep quality in the last month.^16^ We used the Dutch version of the PSQI, which is widely used in the Netherlands and has been used to assess sleep in numerous previous studies on sleep quality on Dutch populations.^21,22^ The total PSQI score is the sum of seven components assessing different sleep aspects, such as sleep latency, usage of sleep medication, and disturbances. All seven components range from 0 to 3 and the total score ranges from 0 to 21, with a higher score indicating more subjective sleep problems. Poor sleep is indicated by a total score of 5 or higher.^16^ The LOAS and NEO participants completed the PSQI at baseline, which means before surgery for the LOAS participants and for the NEO participants when they started filling out surveys. For this study, we compared six of the seven PSQI components measured at baseline (before surgery for the LOAS participants or the first assessment for the NEO participants). PSQI component 4 was omitted from the analyses because, in the LOAS study, a continuous score (number of hours of sleep was categorized instead) was missing and without this continuous score it is not possible to calculate component 4. Therefore, in the current study, the range of the total PSQI score was 0 to 18.

### Statistical analysis

We summarized participant characteristics as mean (SD) or number of observations with percentages for the end-stage hip and knee OA participants and NEO participants separately. We calculated the percentage of individuals per category for every PSQI component except component 4. Furthermore, we calculated the total PSQI scores and determined the medians with IQRs.

We applied multivariable-adjusted linear regression analyses to assess whether end-stage hip or knee OA is associated with the total PSQI sleep score, subjective sleep quality (PSQI component 1, ‘very good’, ‘fairly good’, ‘fairly bad’, or ‘very bad’) and having trouble sleeping due to pain (PSQI question 5i, ‘not during the past month’, ‘less than once a week’, ‘once or twice a week’, or ‘three or more times a week’), compared with the study sample representing the participants without OA. All regression analyses were performed separately comparing end-stage hip and end-stage knee OA with the population controls due to their potentially distinct effects on sleep. In addition, all linear regression analyses were adjusted for the following considered confounding factors: age, sex, smoking, and BMI. These analyses were performed with SPSS Statistics v. 29.0.0 (IBM, USA).

We performed mediation analyses to determine whether the associations between symptomatic end-stage hip or knee OA and subjective sleep quality are mediated by having trouble sleeping due to pain. We estimated the total effect of symptomatic end-stage OA on subjective sleep quality, the direct effect of symptomatic end-stage OA, and trouble sleeping due to pain on subjective sleep quality and the indirect effect mediated by having trouble sleeping due to pain using linear regression models. We assessed whether there was interaction between the exposure and the mediator by including an interaction term between end-stage OA and trouble sleeping due to pain. In all linear regressions we adjusted for potential confounders that were determined a priori (notably, age, sex, smoking, BMI). In addition, we estimated the percentage of the effect that was mediated by the mediator. In case of interaction (p-value_interaction_ smaller than 0.05), we calculated the average causal mediation effect (ACME) and the average direct effect (ADE) for the mean values of the mediator (trouble sleeping due to pain) in the populations with OA and without OA instead. R v. 4.3.1 (R Foundation for Statistical Computing, Austria) with the R package mediation^23^ was used for the calculations. Statistical significance was set at p < 0.05.

To address the difference in age distribution between LOAS and NEO cohorts (notably, LOAS age range 25 to 93 years (mean age 68 years),^19^ and NEO age range 45 to 66 years (mean age 56 years),^18^ we conducted sensitivity analyses in which we restricted all of our analyses to the overlapping age range (45 to 66 years).

## Results

### Baseline PSQI total and component scores

Participants with symptomatic end-stage hip and knee OA reported sleep problems more frequently compared with individuals without OA. The prevalence of individuals with a poor sleep score (total PSQI score > 5) was 54% in the hip and 48% in the knee OA group compared to 21% in the cohort with participants without OA. The median total PSQI score was higher in the end-stage OA groups (6 points (IQR 4 to 9) and 5 points (IQR 3 to 8) for the hip and knee OA groups respectively), compared with 4 points (IQR 2 to 5) in the sample representing individuals without OA (Table II). To analyze the impact of omitting PSQI component 4 in our study, we assessed the total PSQI score of the NEO participants including component 4 leading to a rise of participants reporting a total PSQI above the cutoff of 5 points from 23% to 28%.

**Table II.** Baseline Pittsburgh Sleep Quality Index component scores of the end-stage hip or knee osteoarthritis (OA) patients and Netherlands Epidemiology of Obesity participants without OA.

Variable	End-stage hip OA (n = 922)	End-stage knee OA (n = 870)	Without OA (n = 1,165)
**1. Subjective sleep quality, n (%)**			
Very good	158 (17.1)	180 (20.7)	340 (29.2)
Fairly good	502 (54.4)	506 (58.2)	666 (57.2)
Fairly bad	206 (22.3)	152 (17.5)	148 (12.7)
Very bad	56 (6.1)	32 (3.7)	11 (0.9)
**2. Sleep latency, mins, n (%)**			
≤ 15	225 (24.4)	243 (27.9)	492 (42.2)
16 to 30	315 (34.2)	308 (35.4)	425 (36.5)
31 to 60	217 (23.5)	186 (21.4)	158 (13.6)
> 60	165 (17.9)	133 (15.3)	78 (6.7)
**3. Sleep duration, hrs, n (%)**			
> 7	341 (37.0)	333 (38.3)	466 (40.0)
6 to 7	342 (37.1)	330 (37.9)	603 (51.8)
5 to 6	170 (18.4)	157 (18.0)	73 (6.3)
< 5	69 (7.5)	5 (5.7)	15 (1.3)
**5. Sleep disturbance, n (%)**			
Not during the past month	21 (2.3)	25 (2.9)	120 (10.3)
Less than once a week	552 (59.9)	551 (63.3)	850 (73.0)
Once or twice a week	342 (37.1)	292 (33.6)	118 (10.1)
Three or more times a week	7 (0.8)	2 (0.2)	3 (0.3)
**5i. Sleep disturbance due to pain, n (%)**			
Not during the past month	186 (20.2)	247 (28.4)	1,000 (85.7)
Less than once a week	127 (13.8)	131 (15.1)	62 (5.3)
Once or twice a week	162 (17.6)	169 (19.4)	53 (4.5)
Three or more times a week	447 (48.5)	323 (37.1)	50 (4.3)
**6. Use of sleep medication, n (%)**			
Not during the past month	646 (70.1)	629 (72.3)	1,025 (88.0)
Less than once a week	57 (6.2)	51 (5.9)	55 (4.7)
Once or twice a week	51 (5.5)	61 (7.0)	32 (2.7)
Three or more times a week	168 (18.2)	129 (14.8)	52 (4.5)
**7. Daytime dysfunction, n (%)**			
No problem at all	348 (37.7)	404 (46.4)	650 (55.8)
Only a very slight problem	473 (51.3)	405 (46.6)	415 (35.6)
Somewhat of a problem	90 (9.8)	53 (6.1)	91 (7.8)
A very big problem	11 (1.2)	8 (0.9)	7 (0.6)
Mediam PSQI total score (IQR)*	6 (4 to 9)	5 (3 to 8)	4 (2 to 5)

The percentage of individuals per category was calculated for every Pittsburgh Sleep Quality Index (PSQI) component, except component 4.

* Sum of all included Pittsburgh Sleep Quality Index (PSQI) component scores without component 4.

For all six individual PSQI components, the percentages of participants in the worse sleep score categories were higher for end-stage hip and knee OA patients compared with the participants without OA. With regard to the outcome ‘subjective sleep quality’, patients with hip and knee OA more often reported fairly or very bad quality (28.4% and 21.2%, respectively) compared with the participants without OA (13.6%). Also, 65.1% of the patients with end-stage hip OA and 56.5% of the patients with end-stage knee OA reported ‘trouble sleeping due to pain’ at least once a week, compared with 8.8% of the participants without OA. Furthermore, hip and knee OA patients more often used sleep medication three or more times a week (18.2% and 14.8%, respectively) compared with the control group (4.5%) (Table II).

### The association between end-stage hip and knee OA and total PSQI score, subjective sleep quality, and trouble sleeping due to pain

Both symptomatic end-stage hip and knee OA were associated with a higher total PSQI score (hip OA adjusted difference 2.29 points (95% CI 1.96 to 2.62), knee OA 1.88 points (95% CI 1.53 to 2.23)). In line with this, we found that end-stage hip and knee OA were associated with lower subjective sleep quality (hip OA adjusted difference 0.37 points (95% CI 0.29 to 0.44), knee OA 0.23 points (95% CI 0.15 to 0.32)) and trouble sleeping due to pain (hip OA 1.75 points (95% CI 1.64 to 1.86), knee OA 1.50 points (95% CI 1.38 to 1.62)) (Table III).

**Table III.** Multiple linear regression analyses to assess the association between end-stage hip or knee osteoarthritis with worse sleep outcomes.

Variable	End-stage hip OA, mean difference (95% CI)	End-stage knee OA, mean difference (95% CI)
**Total PSQI sleep score (range 0 to 18)** *		
Univariable linear model	2.26 (2.00 to 2.51)	1.71 (1.46 to 1.96)
Multivariable linear model	2.29 (1.96 to 2.62)	1.88 (1.53 to 2.23)
**Subjective sleep quality (range 0 to 3)**		
Univariable linear model	0.32 (0.26 to 0.38)	0.19 (0.13 to 0.25)
Multivariable linear model	0.37 (0.29 to 0.44)	0.23 (0.15 to 0.32)
**Trouble sleeping due to pain (range 0 to 3)**		
Univariable linear model	1.67 (1.59 to 1.75)	1.38 (1.29 to 1.47)
Multivariable linear model	1.75 (1.64 to 1.86)	1.50 (1.38 to 1.62)

The multivariable regression models are adjusted for potential confounding factors: age, sex, BMI, and smoking.

The mean difference is equal to the regression coefficient (β), for OA.

* Without component 4.

OA, osteoarthritis; PSQI, Pittsburgh Sleep Quality Index.

### The mediating role of trouble sleeping due to pain

The estimated total effect of symptomatic end-stage hip and knee OA on subjective sleep quality, the direct effect of OA and trouble sleeping due to pain on subjective sleep quality, and the indirect effect mediated by having trouble sleeping due to pain are shown in Supplementary Tables i to vii.

We did not observe an interaction between the mediator (trouble sleeping due to pain) and the exposure (symptomatic end-stage OA) in patients with end-stage knee OA. The association was completely (percentage of mediation 123% (95% CI 90% to 191%)) mediated by trouble sleeping due to pain for the end-stage knee OA patients, indicating that the indirect effect (association of OA on trouble sleeping due to pain × trouble sleeping due to pain on subjective sleep quality) is even larger than the total effect (OA on subjective sleep quality). The total, direct, and indirect effects for patients with end-stage knee OA are shown in Supplementary Tables i to vii.

We did observe interaction between end-stage hip OA and trouble sleeping due to pain. Therefore, we also estimated the direct and indirect effects using the mean values of the mediator (trouble sleeping due to pain) separately for individuals with and without symptomatic end-stage OA (data not shown), which showed similar results. The entire total effect is accounted by the mediator with a percentage of mediation of 112% (95% CI 90% to 145%).

### Sensitivity analysis

The sensitivity analyses of LOAS participants with an overlapping age range to the NEO participants can be found in the appendix. We did not observe any unexpected changes regarding the baseline characteristics with comparable means of BMI and proportion of females, increase of percentage of participants smoking, but decrease of percentage living alone or having comorbidities. Overall, a slight increase of the regression coefficients was observed. These results suggest that the association between end-stage hip and knee OA and quality of sleep is even somewhat stronger than our primary analysis showed.

## Discussion

### Key findings

Our study shows that 54% of the patients with symptomatic end-stage hip OA and 48% of the patients with symptomatic end-stage knee OA report poor sleep quality, compared with 21% of the population without OA. In addition, people with symptomatic end-stage hip and knee OA report worse subjective sleep quality and more trouble sleeping due to pain. The association between end-stage hip and knee OA and subjective sleep quality was completely mediated by trouble sleeping due to pain.

To our knowledge, the current study is the first to investigate the association of symptomatic end-stage hip and knee OA and quality of sleep within a large European cohort. Our study population comprising over 1,800 participants with symptomatic end-stage hip or knee OA is a considerably larger than those of previous studies in this field with cohorts existing of 163 to 269 OA participants.^11,24^ Using data from the NEO study also enabled comparing sleep in symptomatic end-stage OA patients with a large representative cohort of the general Dutch population without OA. Besides, we analyzed the data for end-stage hip and knee OA patients separately, which is seldom performed. Such a stratified analysis is clinically relevant given the distinct differences between symptomatic hip and knee OA in clinical presentation, prognosis, and pain experience. Consequently, a stratified evaluation of hip and knee OA cohorts is essential for interpretation of data, drawing clinical relevant conclusions, and making clinical relevant decisions.

When considering our findings, it is important to acknowledge the limitations of our study. The data we used came from patient-reported questionnaires, which are inherently subjective and reflect participants’ perceptions of their own sleep rather than objective measurements like sleep duration or efficiency. While these self-reported measures provide valuable insights into perceived sleep quality, they are limited by individual biases and recall issues, which might have led to information bias. Although objective sleep measures such as actigraphy or polysomnography can provide useful data about sleep, our focus was on the subjective experience of sleep. Perceived sleep quality is closely linked to emotional distress, daytime functioning, and pain perception, and therefore offered the most relevant perspective for exploring the psychological interplay between pain and sleep.

Unfortunately, were unable to calculate sleep efficiency objectively, as the LOAS cohort only collected categorized data on sleep duration, making it impossible to calculate this component (component 4) which had to be omitted from the total PSQI score calculations. As such the total PSQI scores obtained in our study are not directly comparable with total PSQI scores reported in other studies. To estimate the impact of omitting this component we assessed the total PSQI score of the NEO cohort including component 4 leading to a rise of participants reporting a total PSQI above the cutoff of 5 points from 23% to 28%. Furthermore, the preoperative patient-reported pain levels for hip and knee OA patients in the LOAS cohort are also subjective, although the indication for performing THA or TKA depends not only on symptom state but also on level of disability, the degree of radiological OA, as well as the effect of conservative measures.^25^ Pain sensitization may play a role in satisfaction after arthroplasty, since about 10% to 20% of these patients is not satisfied after surgery.^26,27^ Although our results provide insights into the self-perceived difference in sleep quality between end-stage hip and knee OA patients and individuals without OA, another limitation could be the different distribution of characteristics in the LOAS compared with the NEO cohort. Especially the mean age of the NEO cohort was significantly lower than that of the LOAS cohort, which introduces non-positivity in part of the age range. Therefore, we included a sensitivity analysis that only included participants aged within the overlapping age range of the LOAS and NEO cohorts. Our sensitivity analysis showed similar results to our original analysis with a slight increase of the regression coefficients (see Supplementary Tables i to vii.).

Also, all participants from the cohort without OA are from one village in the Netherlands (Leiderdorp), while the participants from the LOAS are more distributed across the Netherlands. Previous research from the NEO^18^ study showed that overall physical functioning of the NEO cohort is better than that of the general population. As physical activity and complaints are associated with sleep,^28,29^ these factors could have influenced the results of the NEO cohort. Another study from the Netherlands, the Rotterdam study,^30^ reported a median and IQR of the total PSQI score of 3 (1 to 5). This median is lower than the current study (4, IQR 2 to 5), which is unexpected, because our study did not include component 4. Other studies in Germany and Korea have reported mean total PSQI scores of 5.00 and 5.63, respectively,^29,31^ which are higher than the current study (4.06). Hence, although we adjusted for several confounders in our study residual confounding remains a limitation, which is inherent to observational studies.

### Previous literature

The results of our study align with and extend previous research on sleep in OA patients.^9,14,24,32,33^ As noted in our introduction, OA is the most common musculoskeletal disorder worldwide, with lifetime risks of 25% for hip OA^1^ and 45% for knee OA.^2^ While prior studies established associations between OA and sleep problems,^10^ our findings specifically quantify this relationship in end-stage OA, demonstrating that 54% of hip OA and 48% of knee OA patients report poor sleep quality compared with 21% of the cohort without OA. Our results confirm earlier observations from Asian studies showing shorter sleep duration in OA patients compared with the general population,^14,15^ and American studies documenting higher rates of sleep problems in those with symptomatic hip or knee OA.^9,11^ One American study found that one in seven individuals with radiological knee OA report more than three nights of restless sleep per week,^34^ which aligns with our more severe findings in end-stage OA patients.

This study advances the literature in several key ways. First, while previous research combined hip and knee OA patients despite their potentially distinct effects on sleep^9^ we analyzed these groups separately. Second, other studies compared sleep quality of patients with different stages of OA without comparing with the general population^9,11^ or only to their healthy relatives,^10^ while we compared with a large sample of the Dutch general population without OA. Third, unlike previous studies from Asia^14,15^ or America our western-European cohort provides valuable regional insights, which is important given documented differences in sleep patterns across these populations.^13^ Fourth, we specifically focused on end-stage OA patients, who experience the most severe pain, offering clinicians a deeper understanding of the pain-sleep relationship at this critical disease stage.

Finally, our mediation analysis establishing that pain fully mediates the relationship between OA and poor sleep quality provides a mechanistic understanding not previously established in the literature, suggesting specific clinical pathways for potential pain interventions.

### Implications for future research

Future research should further investigate preoperative sleep problems in patients with symptomatic end-stage hip and knee OA. It is important for future studies, particularly those with longitudinal designs, to explore whether poor sleep quality prior to surgery is associated with worse postoperative outcomes, including impaired recovery, persistent sleep disturbances, mental health problems, and lower patient-reported outcome measures (PROMs).

Understanding these associations will be critical for developing effective strategies to improve perioperative care. In this regard, we recommend that sleep be considered a relevant variable in the core outcome set for clinical studies involving patients with end-stage OA, given its potential impact on mental health, quality of life, and treatment outcomes.

### Implications for practice

Our current study demonstrates a strong association between end-stage hip and knee OA and poor subjective sleep quality, particularly sleep disturbances related to pain. Since better sleep quality is associated with improved general health and quality of life,^8,35^ these findings have important clinical implications. Our findings highlight the need for a comprehensive approach to managing both pain and sleep issues in these patients without implying that earlier surgery is the solution for sleep disturbances. When managing patients with end-stage OA awaiting joint arthroplasty, orthopaedic surgeons should routinely assess sleep disturbances and consider comprehensive pain management strategies. While our study did not evaluate specific interventions, the OARSI guidelines^36^ suggest limited benefits from opioids, indicating that clinicians should consider evidence-based alternatives such as physical therapy, appropriate exercise programmes, and sleep hygiene education for patients on sometimes extended waiting lists for THA or TKA. This multifaceted approach addressing both pain and sleep may improve patient outcomes during the preoperative period.

Our current study demonstrated a strong association between end-stage hip and knee OA and poor subjective sleep quality, particularly sleep disturbances related to pain. Since better sleep quality is associated with improved general health and quality of life,^8,35^ these findings can have important clinical implications. When managing patients with end-stage OA awaiting joint arthroplasty, orthopaedic surgeons should routinely assess sleep disturbances and consider comprehensive pain management strategies. While our study did not evaluate specific interventions, the Osteoarthritis Research Society International (OARSI) guidelines^36^ suggest limited benefits from opioids, indicating that clinicians should consider evidence-based alternatives such as physical therapy, appropriate exercise programmes, and sleep hygiene education for patients on sometimes extended waiting lists for THA or TKA. This multidisciplinary approach, which addresses both pain and sleep disturbances, may improve patient outcomes during the preoperative period.

In conclusion, our study highlights a significant link between symptomatic end-stage hip and knee OA and poor sleep quality, particularly how pain-related sleep disturbances mediate this association.

Given that improved sleep quality is associated with better overall physical and mental health and quality of life, these findings underscore the importance of routine assessment of sleep disturbances in patients awaiting joint arthroplasty. While our study does not advocate for earlier surgery as a solution to sleep disturbances, it emphasizes the importance of a comprehensive approach that includes pain management and sleep interventions. A comprehensive approach addressing both pain and sleep disturbances may enhance patient outcomes during the preoperative period, especially for those on extended waiting lists for hip or knee arthroplasty.

**Take home message**

- This study demonstrates that poor sleep is highly prevalent in patients with symptomatic end-stage hip and knee osteoarthritis (OA) and that this association is largely explained by pain-related sleep disturbances.

- Clinically, these findings highlight the importance of systematically assessing sleep problems alongside pain in this population. Targeting pain-related nocturnal symptoms may improve both sleep quality and overall quality of life in patients with end-stage OA.

## Data Availability

The datasets generated and analyzed in the current study are not publicly available due to data protection regulations. Access to data is limited to the researchers who have obtained permission for data processing. Further inquiries can be made to the corresponding author.
